# *In ovo* sexing and genotyping using PCR techniques: a contribution to the 3R principles in chicken breeding

**DOI:** 10.1038/s41598-026-40562-y

**Published:** 2026-02-21

**Authors:** C. Dierks, A. Förster, D. Meunier, R. Preisinger, C. Klein, S. Weigend, S. Altgilbers

**Affiliations:** 1 Institute for Laboratory Animal Science and Central Animal Facility, Hannover Medical School, Hannover, Germany; 2 EW GROUP GmbH, 49429 Visbek, Germany; 3 The Roslin Institute and Royal (Dick) School of Veterinary Studies, National Avian Research Facility, University of Edinburgh, Greifswald, UK; 4 Institute of Farm Animal Genetics, Friedrich-Loeffler-Institut – Federal Research Institute for Animal Health (FLI), Greifswald, Germany

**Keywords:** *in ovo* sexing, *in ovo* genotyping, KASP, 3R principles, Biological techniques, Biotechnology, Genetics

## Abstract

Early sex determination and genotyping of chicken embryos is crucial for ethical and resource-efficient animal research. We developed a reproducible workflow using whole genome amplification combined (WGA) with Kompetitive Allele Specific PCR (KASP) and standard endpoint PCR to perform *in ovo* sexing and genotyping from embryonic day 4 (ED4) to ED10. Across four study phases involving various chicken lines—including a genetically modified line, Araucana crossbreeds, and commercial layers (total *n* = 819)—we evaluated methodological feasibility, accuracy, and hatchability. Both PCR methods demonstrated high success rates in yielding results (70–100%) and high identification accuracy (92–100%) throughout all developmental stages tested. While the overall efficiency improved with embryonic age, ED7 was identified as the optimal time point to balance reliable sampling with high hatchability. We conclude that this laboratory-scale, PCR-based protocol provides an accessible and precise method for research laboratories to identify sex and genotype simultaneously. This approach directly supports the 3R principles by preventing the hatching of surplus animals before the assumed onset of nociception (ED13).

**Supplementary Information:**

The online version contains supplementary material available at 10.1038/s41598-026-40562-y.

## Introduction

The chicken has emerged as a popular and valuable animal model, contributing significantly to numerous discoveries in biomedical research^1^. It is particularly well-suited for studying early embryo development, immunology, toxicology, oncology, conservation biology, virology disease mechanisms, and epigenetics^2–6^. The fact that it can be accessed without harming the hen is a major advantage of this model^7^. Although the chicken embryo has often been considered a partial replacement model to mice^8,9^, it is crucial to recognize that chicken embryos may also experience pain^10–12^. This has sparked ethical and political debates, particularly concerning the layer industry’s practice of culling male chicks shortly after hatching, for economic reasons. In response, there has been growing interest in finding alternatives to this practice^13^. Research on nociception and pain perception has contributed to the development of early *in ovo* sexing techniques and the identification of optimal developmental stages for their application^14,15^.

Considering the capacity for pain perception of chicken embryos prior to hatching, several countries have implemented regulations on the destruction of embryonated eggs and the culling of chicks^16^.

In France, the culling of hatched chicks from *Gallus gallus* lines intended for the production of eggs for human consumption is prohibited (R214-17, R214-78 Code rural et de la pêche maritime)^17,18^, but exemptions exist: chicks intended for animal food production and incorrectly sexed chicks may be culled^16–18^.

In the United Kingdom, chicken embryos are not classified as mature vertebrates under the Animals (Scientific Procedures) Act 1986 (ASPA) until day 15 of incubation (embryonic day (ED) 15), which constitutes two-thirds of the incubation period^9,19^. This means that manipulating or culling chick embryos can take place outwith ASPA licensing up to ED14. However, a license is required if manipulated embryos are going to be kept past ED14.

Germany has adopted a distinct approach, driven by the latest findings on pain perception in chicken embryos^10–12^. It bans any procedure, including terminating incubation, that could result in the death of a chicken embryo after ED12. However, this restriction on disposing of embryonated eggs only applies if *in ovo* sexing was performed (section 4c German Animal Welfare Act (Tierschutzgesetz))^20^. If *in ovo* sexing was not conducted, the general ban on culling chicks still applies post-hatch.

Crucially, chicken embryos used for research purposes are not yet protected, unless *in ovo* sexing is performed. The German Animal Welfare Regulation Governing Experimental Animals (Tierschutz-Versuchstierverordnung) only mandates protection for mammalian fetuses from the last third of their normal development before birth^21^. Consequently, procedures involving chicken embryos, such as microinjections, only require approval and notification if the chicks are intended to hatch.

The development of specialized laboratory strains and specific pathogen-free lines has further enhanced the chicken’s utility as a research model^22–25^. Genetically altered lines, such as the iCaspase9 surrogate host chicken^23^, offer promising avenues for reducing the number of animals to address research questions^26^. Careful planning and breeding strategies are crucial to minimize the number of animals required for experiments^27^. Some hatched chicken may not possess the desired traits or sex and can only be used partially in experiments, for example as control animals or for further breeding^28^. Culling an animal without a valid scientific or ethical justification is prohibited by Sect.  17 (1) of the German Animal Welfare Act. The UK Guidance on the operation of ASPA requires researchers to strictly adhere to the replacement, reduction and refinement (3R) principles and use breeding management to minimize surplus animals. In contrast, German and Austrian Animal Welfare Law contain the unique term ‘reasonable cause’ for culling, demanding legal justification for the killing of any animal. While scientific research is generally a ‘reasonable cause’, this justification is not automatically granted for surplus animals that were never used in an experiment. This distinction fuels the ongoing ethical debate and calls into question reduction strategies such as cascade utilization^29^.

*In ovo* sex determination techniques, particularly molecular methods such as Polymerase Chain Reaction (PCR) or hormone analysis, enable early identification of an embryo’s sex or other genetical characteristics from embryonic tissue collected during incubation, including pieces from the chorioallantoic membrane (CAM) or amniotic fluid^30–40^. Recent advances have enabled minimally invasive puncture of the eggshell to collect allantoic fluid, followed by PCR analysis^41–43^.

While industrial systems prioritize high-throughput automation, accessible protocols tailored for research environments remain scarce. Since various physical and biochemical *in ovo* technologies are extensively reviewed elsewhere^14,44,45^ this study aimed to develop a user-friendly protocol using standard laboratory equipment for the simultaneous identification of sex and specific genetic markers via PCR. Early *in ovo* sex determination, which does not require complex equipment, would benefit breeding management, particularly for genetically engineered chicken lines. This capability is crucial for all laboratories committed to the 3Rs principles as it directly enhances animal welfare by allowing the removal of surplus embryos before the assumed onset of nociception (ED13). By providing a robust screening tool, the method mitigates significant issues concerning biosecurity, compliant waste disposal, and excessive animal resource demands. While beneficial globally, this solution is particularly vital for new transgenesis laboratories, especially those in Low- and Middle-Income Countries, which often rely on less-efficient initial breeding methods due to a lack of surrogate host systems^23,46,47^.

## Materials and methods

### Animal experiments and animal care/ethical statements

The study is reported in accordance with the ARRIVE guidelines^48,49^. Sexing chickens before hatch is a common practice in hatcheries in Germany. In the present study, samples from chicken embryos were obtained prior to the assumed onset of nociception (ED13)^10–12^; this did not require an animal experiment application, which is in accordance with the legal framework for animal experiments in Germany (German Animal Welfare Act (TierSchG)^20^ and Animal Welfare Regulation Governing Experimental Animals (TierSchVerV)^21^. The German legislation is based on the overarching EU directive 2010/63/EU. Allantoic puncture and blood sample collection from hatched chicks during the Phase I study were approved by the Animal Care and Use Committee of Lower Saxony (Niedersächsisches Landesamt für Verbraucherschutz und Lebensmittelsicherheit), Oldenburg, Germany (approval number: AZ 33.9-42502-05-10A064)).

### Experimental design

**Fig. 1:**
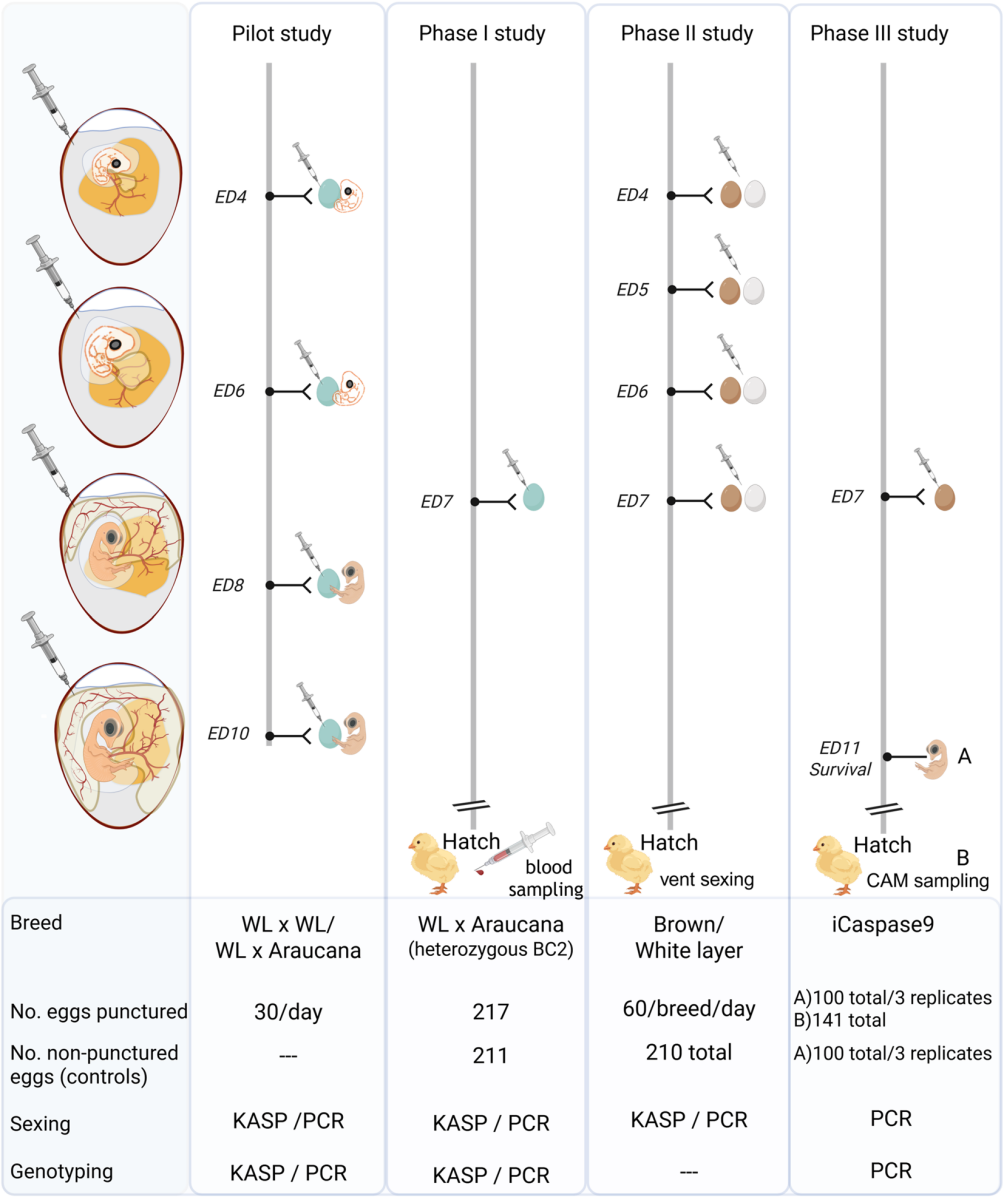
Experimental Workflow for *in ovo* or Allantoic Fluid Collection from Embryonated Eggs. Schematic representation of chicken embryo development from day five (ED4) to eleven (ED10) of incubation, highlighting rapid allantoic vesicle formation and chorioallantoic membrane (CAM) development. The study comprised a Pilot study and three subsequent studies (Phase I, II, III). In each study, eggs were punctured at various time points during incubation to obtain fluid samples for DNA extraction and PCR analysis. In the Pilot study, embryos were dissected after puncture to obtain tissue samples for sex verification. In Phase I and Phase II studies, embryos were allowed to hatch after sampling, and blood samples were collected or vent sexing was performed to verify *in ovo* sexing accuracy. In the Phase III study, eggs from genetically modified embryos were punctured at ED7 and then returned to the incubator until ED11. (**A**) Survival rates were assessed at ED11 post-puncture, and a tissue sample was collected from each embryo. (**B**) From 141 fertilized eggs punctured on ED7, a defined subset was allowed to hatch, and CAM samples were collected for DNA extraction. (Created in BioRender. Altgilbers, S. (2025) https://BioRender.com/gy26oy3).

The study followed a four-stage experimental design to validate *in ovo* sexing and genotyping across different genetic lines and developmental stages, as summarized in Fig. 1.

Pilot Study: This initial phase aimed to identify the optimal time point for fluid collection by testing various embryonic stages (ED4-ED10). The focus was on technical feasibility and DNA yield from a crossbred population (White Leghorn x Araucana).

Phase I: Using the same breeds but progressing to subsequent backcross generations (as detailed in ‘Phase I Study’ Methods section), Phase I evaluated the impact of sampling at ED7 on hatchability in a larger cohort, with all sampled eggs incubated to hatch. In both the Pilot and Phase I, sexing was supplemented with genotyping to identify the blue eggshell trait (marker-assisted introgression).

Phase II: This phase served as a field validation using commercial white and brown layer lines. The objective was to assess sexing accuracy and hatch rates specifically between ED4 and ED7. The analysis was limited to sex detection, as these were standard commercial lines without additional genetic traits.

Phase III: A proof-of-concept study was conducted using a genetically modified line (iCaspase9 surrogate host)^23^. Genotyping was performed alongside sexing (ED7) to verify the transgene status (heterogeneity) for the selective hatching of desired individuals, thereby reducing the number of surplus animals in research.

To guide the selection of appropriate time points for allantoic puncture, an *ex ovo* culture was first set up to assess the spatial extent of the CAM and allantoic sac across different incubation days. Early *in ovo* sampling confirmed that various internal egg fluids contained DNA. To reflect this, the term “*in ovo* fluid” is used for all samples collected before ED7. From ED7 onward, we are confident that the samples represent allantoic fluid.

### *Ex ovo* culture

A shell-less culture protocol was used from ED2 to ED11 as previously described^50,51^. *Ex ovo* culture was initiated with embryos at Hamburger and Hamilton^52^(HH) stage 15–17, corresponding to approximately 65 h of incubation. Embryos in weigh boats were covered with a petri dish lid and incubated without rotation in a fully automated digital incubator (Ova-Easy 100 Advance Series II Cabinet Incubator, BRINSEA PRODUCTS LTD, North Somerset, UK) at 65% relative humidity using a humidity pump (Ova-Easy/TLC Advance Humidity Pump, BRINSEA PRODUCTS LTD, North Somerset, UK). Photographs of embryos and gonads, and allantoic cavity measurements (96–144 h; *n* = 10–15 embryos/incubation day) were acquired by light microscopy using an Olympus SZ61 stereomicroscope equipped with an Olympus EP50 camera and the associated EPView Windows software or iOS App (EVIDENT Europe GmbH, Hamburg, Germany). Images shown in Fig. S1 were captured using an iPhone 13 Pro (12 MP camera), allantoic cavity measurements (168–192 h) were taken manually with a ruler, and scale bars were subsequently set in ImageJ 1.48v/Java 1.6.0_20 (64-bit). Using an insulin syringe (Omnican 40; B. Braun Melsungen AG, Melsungen, Germany), food coloring was precisely injected into the allantoic or amniotic cavity under microscopic control (Olympus SZ61 stereomicroscope).

### *In ovo*/allantoic fluid collection

**Fig. 2:**
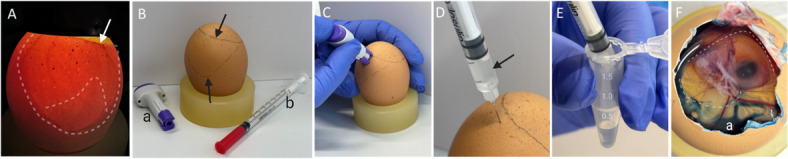
Step-by-Step Demonstration of Allantoic Fluid Collection. (**A**) Fertile brown egg (ED7) candled with an LED light. The white arrow indicates the border between egg contents and air cell at the blunt end of the egg. The white dashed lines delineate a yolk-free area of the egg, with the embryo visible at the bottom of this area, shining through the fluids within the amnion and chorioallantoic sac. (**B**) The air sac and the yolk free area of the egg, as described in (**A**), were marked with a pencil (black arrows); (**a**) single-use lancing devices (ACCU-CHEK, Safe-T-Pro UNO, Roche AG, Basel, Switzerland), (**b**) insulin syringe (Omnican 40; B. Braun Melsungen AG, Melsungen, Germany). (**C**) A small hole was created in the eggshell using a lancing device, positioned close to the border of the air chamber. (**D**) The insulin syringe was used to aspirate the clear allantoic fluid (black arrow) through the previously created hole. For aspiration, the syringe needle was inserted to a depth of about 3–4 mm into the egg. (**E**) The aspirated allantoic fluid was transferred into a tube. (**F**) To demonstrate that the amnion was not punctured during the procedure, an egg was opened at the blunt end after the procedure. Blue food coloring (**a**) was injected through the hole in the eggshell, and no color was observed in the amnion cavity (marked with white dashed lines), confirming that only the allantoic cavity had been punctured (ED11).

Across all study phases, fluid was collected following a standardized protocol (Fig. 2).

The air chamber and embryo location were identified using a LED light egg candler (Fig. 2A; Fig. S1A). Eggs with displaced air chambers, those that were unfertilized, or those showing early embryonic death were excluded from the study (Fig. S1B-D). The eggshell was positioned blunt-end up, and a puncture site was marked approximately 2–5 mm below the air chamber, as previously described^37^(Fig. 2B). To access the embryo, a small hole was created in the eggshell using either an awl, a stainless-steel T-pin (specifically for harder shells), or a sterile, single-use lancing device (ACCU-CHEK, Safe-T-Pro UNO, Roche AG, Basel, Switzerland) (Fig. 2C). Approximately 50 (up to a maximum of 100 µl) microliters of *in ovo* or allantoic fluid was extracted from each egg using an insulin syringe (Omnican 40; B. Braun Melsungen AG, Melsungen, Germany) and transferred to a 1.5 ml Eppendorf tube (Eppendorf SE, Germany, Hamburg) (Fig. 2B-E), stored on ice, and subsequently frozen at -20 °C. If sampling was unsuccessful after two attempts, the egg was discarded from analysis.

### DNA processing, amplification and quantification

DNA was extracted from *in ovo* or allantoic fluids using the REPLI-g Mini Kit (Qiagen, Hilden, Germany) following the manufacturer’s protocol with slight modifications. Samples stored at -20 °C were thawed on ice and centrifuged at 13,000 rpm for 1 min. The supernatant was carefully removed to ensure the non-visible pellet remained undisturbed. This pellet was resuspended in 3 µl of phosphate-buffered saline and stored on ice. Initially, the 3 µl sample was incubated with Buffer D2 for cell lysis and DNA denaturation according to the manufacturer’s instructions. For Whole Genome Amplification, 40 µl of the REPLI-g master mix was added to each sample, containing Phi29 DNA polymerase and exonuclease-resistant random hexamer primers to initiate Multiple Displacement Amplification (MDA)53,54. The reaction was carried out in a heated lid thermocycler (Eppendorf Mastercycler). Due to instrument programming limits, the 16-hour isothermal incubation at 30 °C was performed in 16 consecutive 60-minute blocks. This was followed by an inactivation step at 65 °C for 3 min to terminate enzymatic activity and a final hold at 4 °C. The heated lid temperature was maintained at 70 °C throughout the process.

Representative fluidic samples were stained using Hoechst to demonstrate the presence of cells and/or DNA within the obtained fluid. For Hoechst staining, we resuspended the pellet in 10 µl of Hoechst 33,342 solution (5 µg/ml). This was then transferred to a microscope slide, covered with a coverslip, and evaluated using fluorescence microscopy (Zeiss Axioscope 5 with Colibri 5 LED light and Axiocam 202 mono), employing a multi-band pass filter (FS 90 HE LED; Carl Zeiss GmbH, Oberkochen, Germany) and 385/30 nm BP excitation for visualization.

For tissue samples, the DNeasy Blood & Tissue Kit (Qiagen) was used. Blood samples were collected on medium filtration capacity filter paper cards^55^(FTA^®^ card, Schleicher & Schuell GmbH, Whatman, Inc., Maidstone, UK). DNA was then extracted using a pronase digestion protocol as described^56^. CAM and other embryonic tissue were digested by overnight incubation with proteinase K (10 mg/ml) in lysis buffer (100 mM Tris-HCl, 100 mM NaCl, 100 mM EDTA, 1% SDS) at 56 °C. After removal of precipitated proteins using saturated NaCl solution and centrifugation, DNA was precipitated with 100% ethanol. The resulting DNA pellet was washed twice with 70% ethanol, air-dried, and dissolved in 30 µl H_2_O. DNA concentration was measured using a FLUOstar Omega (BMG Labtech, Ortenberg, Germany) or Qubit 4 fluorometer (Thermo Fisher Scientific, Waltham, USA) respectively. The concentration of DNA amplified with the REPLI-g Mini Kit was determined using fluorescent dye-based quantification assays, either the Quant-iT PicoGreen dsDNA Assay Kit (Thermo Fisher Scientific, Waltham, USA) or the Qubit 1X dsDNA HS Assay Kit (Invitrogen, Waltham, USA). DNA from WGA samples was visualized on a 0.8% agarose gel (Agarose NEEO ultra-quality, Carl Roth GmbH + Co. KG, Karlsruhe, Germany) in 1x TBE buffer. Fluorescent staining of nucleic acids in agarose gel was done with ROTI GelStain (Carl Roth GmbH + Co. KG, Karlsruhe, Germany). Signals were visualized using a Fusion SL4-3500.WL (Vilber, Eberhardzell, Germany) UV transilluminator and FusionCapt V15.18 software with a 1.5-second exposure time.

REPLI-g DNA that failed to give a PCR result were purified by ethanol precipitation and the PCR reaction was repeated.

### Sexing and genotyping by PCR

#### Sexing

Sex determination was performed using a multiplex PCR method modified from Fridolfsson and Ellegren (1999) and a KASP assay (LGC Genomics GmbH, Germany, Berlin), which is a fluorescence resonant energy transfer (FRET) cassette based assay. The KASP assay utilizes allele-specific forward primer to target an A/G difference in exon 17 of the conserved chromodomain helicase DNA binding protein 1 (*CHD1*) gene on the W- and Z-chromosomes (*CHD-Z*/*CHD-W*; Table S1) as described^57^. The multiplex PCR assay exploits an intronic length polymorphism between the CHD-Z and CHD-W^58^. 10 ng of DNA was added to the PCR master mix for each sample.

#### Genotyping

Samples were genotyped for the blue egg (Pilot study and Phase I study) and the iCaspase9-GFP-*DAZL*^23^ loci (Phase III study).

The causal mutation of dominant blue egg shell color in the Araucana breed is a 4.2 kb retroviral insertion (EAV-HP) on chromosome 1 upstream of *SLCO1B3* at 65.22 Mb, which was detected by PCR according to Wragg et al. (2013)^59^ and previously described by our group^60^. Samples from Phase III Study were genotyped for the iCaspase9-GFP integration site in the DAZL locus to distinguish between wildtype, heterozygous, and homozygous carrier animals as described by Ballantyne et al. (2021). PCR amplification was performed using Promega GoTaq Polymerase (Promega, Madison, USA) under the following conditions: initial 95 °C for 2 min, 94 °C for 30 s, 64 °C for 30 s, 72 °C for 30 s for 35 cycles and a final extension of 72 °C for 5 min. Reaction products were resolved using a 1.5% ultrapure agarose (Invitrogen, Waltham, USA) gel electrophoresis run at 80 V for 45 min in 1xTBE buffer and visualized using a UV transilluminator. 10 ng of DNA was added to the master mix for each sample.

Each KASP reaction contained about 20–50 ng template DNA, KASP V 4.0 2x Master mix standard ROX (LCG Genomics, Berlin, Germany) and KASP-by-Design assay mix (LGC Genomics). Standard KASP thermal cycling conditions, as described in LGC protocols, were performed using an Eppendorf Mastercycler (Eppendorf, Hamburg, Germany). KASP PCR cycling (blue eggshell) consisted of an initial denaturation step at 94 °C for 15 min, followed by 10 touchdown cycles (94 °C for 20 s, 61 –55 °C for 60 s, with a 0.6 °C decrease per cycle). This was followed by 26 additional cycles at 94 °C for 20 s and 55 °C for 60 s. Following amplification, microplates were analyzed with FLUOstar Omega (BMG Labtech, Ortenberg, Germany). Fluorescence signals were detected at excitation/emission wavelengths of 485/520 nm for FAM-labelled-FRET-cassettes, 530/560 nm for HEX-labelled-FRET-cassettes, and 584/620 nm for ROX standard.

## Methodological approach and study phases

### Statistical analysis

Statistical analyses were performed with the R statistical software (v.4.0.0, R Core Team 2023). Hatchability and mortality rates were compared using two-sided Fisher’s exact tests, with effect sizes reported as odds ratios (OR) and 95% confidence intervals (95% CI). The Cochran-Armitage trend test evaluated the correlation between intervention timing (ED4–ED7) and hatching success. Growth data are presented as means ± SD. The level of significance was set at *p* < 0.05. Descriptive statistics are presented as percentages, rounded to the nearest whole number.

#### Phase I study

The hens and roosters used for egg production were obtained from the second backcross of Araucana with WL. Over two generations, heterozygous roosters for the blue eggshell allele were backcrossed to WL hens. For the present study, hens that were heterozygous for the dominant blue egg-laying trait were inseminated with sperm from heterozygous roosters of the backcross generation. 480 fertile eggs were incubated in an auto-rotating incubator (PETERSIME NV, Zulte, Belgium) under standard conditions (see Pilot study). At ED7 (184 h), 50 µl of fluid were extracted from half of the incubated eggs (217 total) and subjected to sexing and genotyping by Kompetitive Allele Specific PCR (KASP) and PCR. During the second candling (≈ ED18), eggs showing embryonic loss were sampled for tissue collection (embryo retrieval) to verify the *in ovo* sexing and genotyping results using a KASP assay. For samples with discrepancies between allantois and blood/tissue typing, sex determination or genotyping was confirmed by standard endpoint PCR. The remaining 211 eggs served as non-punctured controls to assess hatch rates and were temporarily kept outside the incubator for the same length of time as eggs submitted to sampling. After hatching, blood samples were collected from the 7-day-old chicks that had been subjected to *in ovo* sexing. Blood samples were collected from the *Vena metatarsalis plantaris superficialis medialis* using a lancet and collected on filter paper (Fig. S3), and subjected to sexing and genotyping by KASP after DNA extraction.

#### Phase II study

450 brown eggs from 63-week-old hens, and 450 white eggs from 42-week-old hens, sourced from commercial layer flocks, were collected. White eggs were stored for six days and brown eggs were stored for nine days before incubation was initiated. All 900 eggs were then incubated under standard conditions in an auto-rotating incubator (EMKA Hatchery Equipment B.V., Kuurne, Belgium) at Lohmann Breeders GmbH.

From ED4 to ED7, approximately 50 µl of fluid was collected from at least 60 eggs per line per day; exact incubation lengths are listed in. Samples were subjected to sexing and genotyping by KASP assay and standard endpoint PCR. Eggs were candled at ED4, ED7, and again around ED18, a common practice in poultry husbandry before transferring eggs from the setter to the hatcher. After hatching, chicks were sexed visually (vent sexing) by an experienced chick sexer, a standard procedure in commercial poultry facilities. Non-punctured, untreated eggs obtained from 63- and 42-week-old hens, respectively, were used as hatching controls. The brown layer groups represented a cumulative total from three hatches, while the white layer control groups represented a cumulative total from five hatches.

#### Phase III study

A total of 100 iCaspase9 eggs, collected from 43-46-week-old hens over a 7-day period, were incubated for 190 h under standard conditions in a fully automated digital incubator (Ova-Easy 100 Advance Series II Cabinet Incubator, BRINSEA PRODUCTS LTD, North Somerset, UK). Eggs were collected over 7 days. The eggs were obtained from natural mating between: (1) a heterozygous iCaspase9 rooster and iCaspase9 homozygous and heterozygous hens, and (2) a homozygous iCaspase9 rooster and wild-type L68 hens (New Hampshire descent). After sample collection, eggs were returned to the incubator until ED11, when survival rates post-puncture were assessed. Embryos were then dissected and tissue samples were frozen at -20 °C for subsequent sexing and genotyping by PCR. The experiment was repeated using 141 iCaspase9 fertilized eggs. Eggs were collected and subsequently stored at 15 °C with 75% relative humidity for a maximum of three weeks. Allantoic fluid was collected from all 141 eggs by puncturing at ED7. Subsequently, 29 eggs of the desired sex and genotype were incubated to hatch. Eggs were moved to the hatcher (Ova-Easy Advance Series II Hatcher, BRINSEA PRODUCTS LTD, North Somerset, UK) at 18 days and 4 h and incubated at 37.3 °C and 65–70% relative humidity. CAM samples were collected from all eggs in the hatcher. Chick quality was assessed using the Pasgar©Score and Tona Score^61,62^.

#### Pilot study

White Leghorn (WL) hens were inseminated with sperm from WL or homozygous blue egg layer Araucana roosters. Eggs were stored for up to six days at 15 °C. For incubation, eggs were placed in an automatic turning incubator (BRUJA, Brutmaschinen-Janeschitz GmbH, Hammelburg, Germany). The incubator was set to maintain a temperature of 37.8 °C and a humidity level of 50–55%, with eggs being turned 45° at regular intervals. Thirty fertilized eggs were incubated for a full 4 (96 h), 6 (144 h), 8 (192 h), or 10 (240 h) days. The first day of incubation was assigned as ED0. Following collection of *in ovo* fluid, embryos were dissected and tissue samples were collected from all embryos and frozen at -20 °C for subsequent DNA extraction to confirm the results obtained from analysis of the *in ovo* fluid samples. In addition, embryonic gonads from ED10 embryos were visually examined for sex identification (Fig. S2A-D).

## Results

### Allantois formation in shell-less culture system

**Fig. 3:**
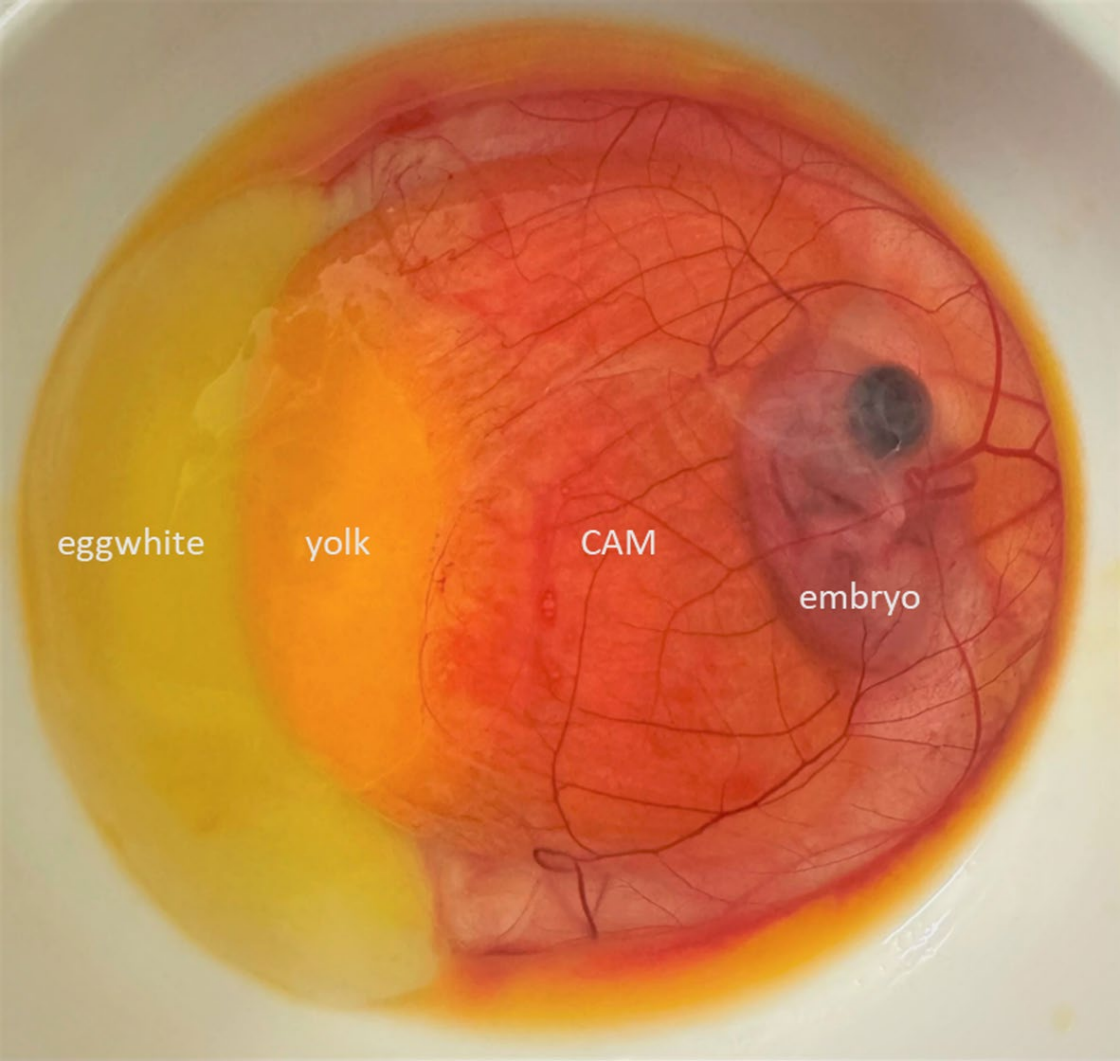
Chicken Embryo and CAM Development at ED9. The highly vascularized chorioallantoic membrane (CAM) encloses the embryo within the amnion and partially covers the yolk sac from all sides at ED9 (indicated by white dashed lines). The CAM is still intact in the depicted image; however, it typically tears when opening the egg shell due to its close connection to the inner shell membrane.

To visualize embryonic development and assess the size of the amnion, allantois and CAM at the time of sample collection, an *ex ovo* culture system adapted from existing methodologies^50,51^ was used (Fig. S4). At 65 h of incubation (ED2; Fig.S4A), the amnion, which will ultimately enclose the embryonic body, was not yet fully closed. The amniotic fold remained visible, positioned approximately at the level of the posterior vitelline artery (Fig. S4B). By ED3 (72 h; Fig. S4C), the amnion appeared as a small vesicle, originating as an evagination from the endodermal hindgut (Fig. S4D). At ED4 (96 h), the allantois presented as a vesicle, roughly equivalent in size to the embryo’s midbrain (Fig. S4E). At ED4 (116 h; Fig. S4F-H), the allantois was observed to have a mean diameter of 1.67 cm (SD = 0.21 cm), while the embryos themselves had a mean size of 1.47 cm (SD = 0.09 cm; HH stage 25–26, *n* = 10). At ED6 (144 h; Fig. S4I-K), the size of the allantois increased to 3.1 cm (SD = 0.39 cm) with a mean embryo size of 2.4 cm (SD = 0.48 cm; HH stage 28–29, *n* = 10). By ED7 (168 h; Fig. S4L-N), the allantois reached a diameter of 4.9 cm (SD = 0.57 cm; HH stage 30–32, *n* = 10). This allantoic sac further expanded to 6.6 cm (SD = 0.64 cm; HH stage 33–34, *n* = 10) at ED8 (192 h; Fig.S4O-Q), by which time it largely covered the yolk in most of the embryos. At ED9 (240 h; Fig. S4R-T), the allantois entirely covered the yolk and extended beyond it (HH stage 35–36, *n* = 10), as also illustrated in Fig. 3. By ED10 (264 h; Fig.S4U) and ED11 (280 h; Fig. S4V), the allantois contained a greater volume of fluid in all inspected embryos (HH stage 37–38, *n* = 10). In all photographs taken, the embryo is clearly visible within the amniotic cavity, which enlarged concurrently with the growth of the embryo (Fig. S4).

Given the findings described above (Fig. S4E-N), from here on samples collected between ED4 and ED7 of incubation will be referred to as *in ovo* fluid, while samples collected after ED7 of incubation will be referred to as allantoic fluid.

### Pilot study

Across all incubation days (ED4, ED6, ED8, and ED10), 17.5% of the initial shell punctures were unsuccessful, requiring more than one attempt to collect *in ovo* or allantoic fluids. However, sample collection was successful for all eggs (*n* = 30 per group). A closer examination of the fluids collected at ED4 and ED7 revealed the presence of very few stainable cells in the sediment (Fig. S5). Direct use of allantoic fluid, simple cell pellet boiling, and standard DNA extraction kits failed to provide measurable DNA yields or consistent PCR results.

WGA yielded high-quality DNA of high molecular weight (Fig. S6). Mean DNA concentration at ED4 was 65.9 ng/µl and remained stable between 72.1 and 75.9 ng/µl from ED8 to ED10 (Table S2). Three samples from ED4 (96 h) showed no detectable DNA on agarose gel. Multiplex PCR-based sexing and genotyping and subsequent gel electrophoresis were successful from the earliest sampling point (ED4) onwards. However, the success rate was lowest at ED4, with 80% for sexing and 67% for genotyping. At ED6, ED8, and ED10 success rates reached 100% for sexing and 93%-100% for genotyping, respectively (Table S2).

**Fig. 4:**
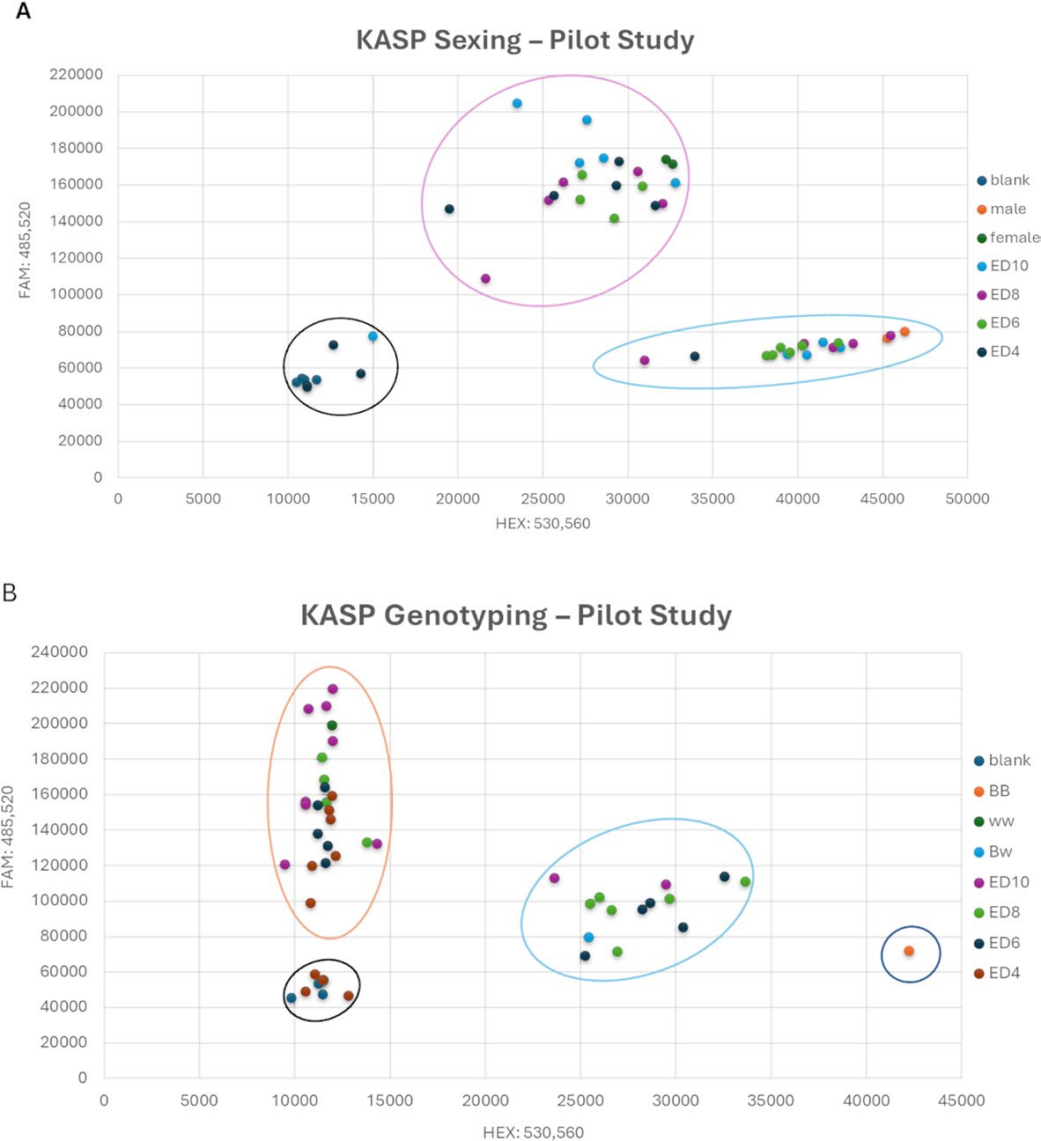
Fluorescence-based Allelic Discrimination using DNA from *in ovo* or Allantoic Fluid. Representative cluster plots for the Kompetitive allele specific PCR (KASP) sexing and genotyping assay (Pilot study) using whole-genome amplified (WGA) DNA extracted from *in ovo* or allantoic fluid. (**A**) Sexing cluster plot: The female-specific forward primer signal (FAM) is plotted against the male-specific forward primer signal (HEX). Distinct clusters represent females (circled in pink) and males (circled in blue), including samples collected at ED4, ED6, ED8 and ED10. The plot also includes reference samples for validation, as well as a no-template control (NTC) cluster (circled in black). The clear separation of these clusters demonstrates the successful differentiation between sexes. A few samples failed to amplify (clustering with NTC) and were subsequently re-analyzed. (**B**) Genotyping cluster plot: The plot displays three distinct clusters: BB (homozygous blue, circled in dark blue), Bw (heterozygous blue, circled in light blue) and ww (wild-type, circled in orange). No-template controls are also shown and circled in black. Reference samples (blood-derived) are labeled as BB, Bw, and ww, while the test samples from ED4, ED6, ED8, and Ed10 are labeled accordingly.

For KASP-based sexing and genotyping, the lowest success rates were observed at ED4 70% and 73%, respectively. Succes rates were significantly higher at ED6 to ED10 (93–100%, Table S2). Sexing and genotyping were successful for all control embryonic tissue samples. Discordant results were observed for sexing and genotyping in two fluid samples from ED6 when compared to the results from the control samples. For ED10 control samples, sex was additionally verified by macroscopic examination of the developing gonads (Fig. S2A-D), confirming the sex previously determined *in ovo*. The results of the allelic discrimination assay, which was used for sexing and genotyping the blue egg allele, are exemplified by the cluster plot in Fig. 4.

#### Phase I study

Of the 480 eggs in the study, 52 eggs were excluded prior to allantoic fluid collection (39 were unfertilized and 13 embryos were determined not-viable at the first candling at ED7). Of the remaining 428 eggs, 217 were sampled at ED7 (184 h), while 211 served as control to assess the impact of egg puncture on hatchability. 28% of the initial shell punctures were unsuccessful, requiring more than one attempt to collect allantoic fluid. The eggs were candled at ED18 to count and remove those with dead-in-shell embryos, and non-hatched chicks were also examined to determine the day of death. Two embryos in each group (control and sampled/punctured) were identified as early embryonic losses (≤ ED 7), having been overlooked at the first candling at ED7 (Table 1). Four embryos in the control group and eleven in the sampled/punctured group were classified as mid-embryonic death (ED7-ED12), occurring shortly after sample collection (Table 1). Most dead embryos were categorized as late embryonic deaths (ED13-ED20), with the highest number of deaths occurring between ED18-ED20. Late embryonic deaths totaled 24 in the sampled group and 13 in the control group. The mid and late embryonic mortality was higher in the sampled/punctured group (16%) compared to the control group (8%). In the control group, 92% (*n* = 192/209) of viable embryos at ED7 yielded healthy hatchlings, compared to 84% (*n* = 180/215) in the sampled/punctured group (Table 1). The difference in the percentage of live chicks was statistically significant (*P* = 0.012; OR = 0.46, 95% CI: 0.23–0.87). Further analysis of embryonic death revealed that the difference in mid-embryonic death (Table 1; sampled/punctured: 5%, *n* = 11/215 vs. non-sampled/non-punctured: 2%, *n* = 4/209) was not statistically significant (*P* = 0.113; OR = 2.76, 95% CI: 0.80-12.07). Similarly, the difference in late embryonic death (Table 1; sampled/punctured: 11%, *n* = 24/215 vs. non-sampled/non-punctured: 6%, *n* = 13/209) also did not reach statistical significance (*P* = 0.085; OR = 1.89, 95% CI: 0.90–4.17).

**Table 1:** Hatching Results for Phase I study.

Group	Total number of eggs	Number of unfertilized eggs or early losses (< ED7)*	Number of viable embryos at ED7	Number of mid-embryonic deaths (ED7-12)**	Number of late embryonic deaths (ED13-20)**	Number of unhatched eggs (ED7-20)**	Number of hatched chicks	Hatchability**	Number of chicks lost post-hatch	Number of healthy hatchlings**
Sampled/punctured	217	2 (1%)	215	11 (5%)	24 (11%)	35 (16%)	180	84%	1 (0.5%)	179 (83%)
Controls	211	2 (1%)	209	4 (2%)	13 (6%)	17 (8%)	192	92%	2 (1%)	190 (90%)
Total	428	4 (1%)	424	15 (4%)	37 (9%)	52 (12%)	372	87%	3 (0.8%)	369 (87%)

Statistical comparisons between the sampled/punctured and control group were performed using Fisher’s exact test. Significant differences (*P* < 0.05) are discussed in the text, including Odds Ratios (OR) and 95% confidence intervals.

*Calculated based on the total number of eggs.

**Calculated based on the number of viable embryos at ED7 (sampled/punctured at ED7).

215 samples were used to validate the accuracy of sexing and genotyping from allantoic fluid DNA subjected to both endpoint and multiplex PCRs with gel electrophoresis and fluorescence-based KASP assays. Results were compared with KASP assay outcomes from DNA extracted from blood of corresponding hatched chicks and embryonic tissue for non-viable embryos.

Standard PCR achieved a success rate of 99.5% for both sexing and genotyping. KASP assays were 100% successful, although 18% of sexing and 5% of genotyping samples required a repeat run due to initial clustering issues (Table 2). Comparison with control tissue demonstrated high accuracy across all methods. KASP sexing from AF was 99% accurate (2 mismatches), while KASP genotyping reached 95% (10 mismatches). Standard PCR on AF showed slightly higher genotyping concordance with the control (97%) than KASP. Direct comparison between PCR and KASP from AF samples resulted in 99% sexing and 98% genotyping concordance (Table 2).

**Table 2:** Sexing and EAV-HP-Insertion Genotyping Success and Accuracy Rates (Phase I study – ED7).

Sample size total/ relative	Number of successful Sexing (PCR)	Number of successful Sexing (KASP)	Number of Concordance Sexing KASP (AF) vs. PCR (AF)	Number of Concordance Sexing AF (KASP) vs. control (KASP)	Number of Concordance Sexing AF (PCR) vs. control (KASP)	Number of Successful Genotyping (PCR)	Number of Successful Genotyping (KASP)	Number of Concordance EAV-HP Genotyping KASP (AF) vs. PCR (AF)	Number of Concordance EAV-HP Genotyping AF (KASP) vs. control (KASP)	Number of Concordance EAV-HP Genotyping AF (PCR) vs. control (KASP)
215	214 (99.5%)	215 (100%)	213 (99%)	213 (99%)	213 (99%)	214 (99.5%)	215 (100%)	211 (98%)	205 (95%)	208 (97%)

AF= Allantoic fluid. control= blood from hatched chicks or tissue from non-hatched chicks but punctured eggs.

#### Phase II study

Most egg fluid samples obtained at ED7 were clear, while samples collected from ED4 to ED6 were frequently cloudy and yellowish (Fig. S7). Only one sample appeared to be bloody (Table 3). Success rates of fluid collection varied depending on the day of incubation. Remarkably, at ED6, up to 25% of the eggs were excluded because fluid could not be successfully extracted despite numerous puncture attempts. However, at least 60 samples from this trial (~ 240 total) were successfully punctured.

**Table 3:** Sample Data for Phase II Study (Chick Sexing ED4-ED7).

Chicken Line	Sampling day	Incubation length (h)	Total no. of samples	Nature of sample			Volume collected			Number of hatched chicks	Hatchability*
				clear	cloudy/ yellowish	bloody	~ 50 µl	~ 25 µl	≥ 75 µl		
B	ED4	108	60	9	51	0	53	2	5	31	52%
W		109	60	6	54	0	51	0	9	54	90%
B	ED5	134	60	10	50	0	36	4	20	47	78%
W		135	60	9	51	0	51	2	7	51	85%
B	ED6	155	60	39	21	0	41	7	12	44	73%
W		156	60	48	12	0	34	8	18	57	95%
B	ED7	180	60	58	1	1	51	2	7	52	87%
W		181	60	59	1	0	49	2	9	58	97%

B= Brown layer, W= White Layer; *Hatchability was calculated based on the total number of samples. Cochran-Armitage trend test indicated a highly

significant chronological increase in hatchability for the brown layer line *(P* = 0.0001), while the white layer line showed consistent resilience across all timepoints (*P* = 0.060).

Following sample collection at ED4, brown eggs exhibited a hatch rate of 52%, while white eggs showed a higher rate of 90% (Table 3). After sample collection at ED5, the hatch rates increased to 78% for brown eggs and reached 85% for white eggs. Sample collection at ED6 led to a slight decrease in the hatch rate for brown eggs (73%), while the hatch rate for white eggs remained high (95%). Finally, following sample collection at ED7, the hatch rates were 87% for brown eggs and 97% for white eggs, the highest rates observed in the study (Table 3). To evaluate the effect of the timing (ED4-ED7), a Cochran-Armitage trend test was performed. A highly significant positive trend in hatchability was observed for the brown layer line (*P* = 0.0001), with hatching rates increasing from 52% at ED4 to 87% at ED7. The white layer line exhibited high resilience across all stages, starting at 90% hatchability at ED4 and reaching 97% at ED7; due to this high baseline, the upward trend for this line did not reach statistical significance (*P* = 0.060).

The overall hatch rate (174/240) for *in ovo* sexed brown eggs was 73%, with a sex ratio of 47% females and 53% males (Table S3). White eggs sexed *in ovo* achieved a hatch rate of 92% (220/239), with a balanced sex ratio of 50% males and 50% females. In these single-hatch groups, unfertilized eggs were excluded prior to sampling. When considering the net yield, the female hatch rate (number of female chicks per total eggs set) was 34% for the brown layer single hatch and 46% for the white layer single hatch. In the flock control groups, incubated simultaneously, the unfertilized egg rate was 26% for brown eggs and 6% for white eggs. This led to a hatch rate of 63% (48% females and 52% males) for brown and 92% (46% females and 54% males) for control white eggs, resulting in a net female hatch rate of 22% and 40%, respectively. Routine hatchery data, derived from a sum of three separate hatches for brown layers and five separate hatches for white layers, demonstrated female hatch rates of 37–41% and 44–47%, respectively. These routine groups exhibited unfertilized egg rates of 12–16% for the brown line and 2–7% for the white line.

KASP sexing demonstrated an overall success rate of 92% across all incubation days (Table 4). Success rates varied by egg type and incubation day: at ED4, KASP achieved 77% success for brown eggs and 87% for white eggs. By ED5, success rates increased to 91% for brown eggs and 86% for white eggs. ED6 yielded further gains, with 98% success for brown eggs and 93% for white eggs. At ED7, the success rate reached 96% for brown eggs and 100% for white eggs. While the overall concordance between KASP results and vent sexing post-hatch was 98% across all incubation days, concordance was slightly lower for both brown and white chicks at ED4 and ED7 (92–96%; Table 4).

**Table 4:** Success Rates of Sex Determination using KASP Assay on *In Ovo* Fluid or Allantoic Fluid Samples (Phase II study).

Chicken Line	Sampling Day	Incubation length (h)	Number of Hatched & Sexed Chicks	Number of Successful KASP Sexing	Concordance KASP & Sexer (*N*)
B	ED4	108	31	24 (77%)	23 (96%)
W		109	54	47 (87%)	45 (96%)
B	ED5	134	47	43 (91%)	43 (100%)
W		135	51	44 (86%)	44 (100%)
B	ED6	155	44	43 (98%)	43 (100%)
W		156	57	53 (93%)	53 (100%)
B	ED7	180	52	50 (96%)	46 (92%)
W		181	58	58 (100%)	56 (97%)
Total	ED4-7	108–181	394	362 (92%)	353 (98%)^*^

B= Brown layer, W=White layer, KASP: Kompetitive allele-specific PCR; Sexer: Sex determination by visual inspection; KASP assay sexing was performed only on samples from live, sexed chicks. *Mean %.

#### Phase III study

Across all experimental groups, 100 iCaspase9 eggs were punctured at ED7, of which 26% were punctured at least twice to aspirate fluid. The aspirated fluid was bloody in four samples, clear in 65 samples, and yellowish in 31 samples. The survival rates at ED11 ranged from 88 to 94% (Table S4). The cumulative survival rate for the punctured group was 92% (*n* = 92/100). To analytically verify the safety of the intervention, this was compared to a simultaneous untreated control group (96% survival; *n* = 96/100). While Fisher’s Exact Test showed no significant difference in cumulative embryonic death (*P* = 0.369; OR = 0.48; 95% CI: 0.12–1.66), the temporal distribution of deaths differed. 100% of embryonic death in the control group occurred prior to ED8, whereas all deaths in the punctured group occurred post-puncturing (ED8–ED11). Specifically, four of the eight deaths in the punctured group exhibited blood in the amniotic cavity at ED9.

Sexing and genotyping results for the iCaspase9-GFP transgene were obtained for all aspirated samples, with a 99% concordance rate compared to tissue analysis (Table S4). The male to female embryo ratio, approximately 50:50, along with the identified genotypes for each trial, are presented in Table S4. As expected, based on the breeding scheme, most embryos were identified as heterozygous iCaspase9 carriers.

The second experimental phase, which included incubation to hatch, involved a total of 177 iCaspase9 eggs. Of these, 141 were confirmed as fertilized by ED7, three died prior to ED7, and the remainder (*n* = 33) were unfertilized. Of the 141 allantoic fluid samples collected at ED7 and initially tested for sexing and genotyping by PCR, 19 did not yield results. Following purification, all but three of the samples produced results (98%). Three mismatches were observed between the allantois and control tissue samples, two for genotyping and one for sexing (Table S5).

29 iCaspase9 eggs of the desired sex and genotype were selected for full-term incubation. All selected eggs were confirmed viable by candling at ED18, and subsequently transferred to the hatcher. A total of 25 chicks successfully hatched. One chick died two days post-hatch due to poor development and open navel at hatch. The remaining chicks were healthy and developed normally (Fig. S8). Specifically, the 29 eggs chosen for hatching comprised: one heterozygous and five homozygous iCaspase9 males, and seven wild-type, 15 heterozygous and one homozygous iCaspase9 females. Of these, one heterozygous and three homozygous males, and seven wild-type and 13 heterozygous females successfully hatched and survived (24 chicks in total: 4 males and 20 females). One heterozygous female chick died in week four (unknown cause). Examination of the five non-hatched chicks showed that they were correctly positioned and developed within the egg but had not initiated internal pipping into the air chamber. Sex and genotypes were confirmed by PCR analysis of allantoic fluid and corresponding post-hatch CAM tissue samples (Fig. S9). Egg weight loss at ED18 averaged 12% (SD = 2%) compared to initial egg weight. The mean hatch weight for chicks of all sexes and genotypes was 41.7 g (SD = 3.3 g). Chicks reached a mean body weight of 253.9 g (SD = 31.4 g) by four weeks and 581.6 g (SD = 61.9 g) by six weeks of age. Their growth performance matched expectations when compared to the parental iCaspase9 chicks (Fig. S10).

## Discussion

In this study, we successfully developed a reliable and user-friendly method for *in ovo* sexing and genotyping of different chicken lines used for research purposes. Our results confirmed the feasibility of *in ovo* sexing and genotyping from ED4 (96 h of incubation) to ED10 (240 h of incubation), and identified an optimal window for allantoic fluid collection which begins at ED7 for maximal reliability, minimal embryonic impact and sufficient hatchability. The choice of ED7 balances this optimal performance window with the requirement to provide sufficient time for DNA analysis before the assumed onset of first signs of embryonic nociception (ED13)^10–12^.

Our shell-less culture system demonstrated the rapid development and expansion of the allantois, a finding consistent with observations in dynamic 3D magnetic resonance imaging (MRI) studies^63^. In early stages (ED2-ED6), the allantois remains a small vesicle, making targeted puncture through the eggshell with a short cannula exceptionally challenging. This is reflected by our observation that fluid collected prior to ED7 was predominantly yellowish rather than clear. We propose that fluid aspirated prior to extended allantois and CAM formation (< ED7) originates from the sub-embryonic fluid (SEF), located within the yolk sac beneath the embryo^64^. While DNA extraction and subsequent sex/genotype determination was feasible with this early fluid, success rates were lower between 96 and 144 h of incubation (ED4-ED6) compared to later stages.

Our observations are supported by the volume dynamics described in the literature. The total SEF volume in the chicken egg reaches a maximum of 13 ml at ED6 and is subsequently reabsorbed until ED15^64,65^, aligning with our data that ED6/ED7 appears to be a turning point. Empirically, this transition is reflected in the high rate of challenging aspirations, which in our study required multiple attempts in 18–28% of eggs across all of our studies. The difficulty in aspirating fluid peaked at ED6 (155–156 h), resulting in 25% of eggs being rejected at this time point during the Phase II study. We suggest that this critical time point reflects the transition from SEF to allantoic fluid, a stage where the developing allantoic sac contains less targetable fluid. This aligns with external reports that sample collection at early stages results in the highest rate of embryo death, likely due to the increased difficulty of the procedure^42^.

Given the crucial role of SEF in early embryonic viability, including potential contributions to acid-base regulation and tissue hydration^66^, our data suggest that the risk of compromising embryonic development through fluid extraction decreases as incubation progresses. Higher hatch rates in eggs punctured later in development compared to those punctured earlier (Phase II study) are an indicator of this reduced risk. While the white layer line demonstrated consistent resilience across all timepoints (*P* = 0.060, Cochran-Armitage trend test), the brown layer line exhibited a significant positive trend in hatchability (*P* = 0.0001), identifying early developmental stages as more sensitive for this specific group.

Previous studies have shown that allantoic fluid volume sharply increases from ED7 onwards, following a parabolic-shaped profile that peaks at ED12^63,64^. We hypothesize that this augmented fluid volume acts as a protective buffer, minimizing the potential for damage to embryonic development and extraembryonic membrane expansion. We visually confirmed that, at ED7, the fluid was exclusively present in the allantoic sac, not in the amnion. This accumulation is consistent with the allantois serving as a repository for embryonic kidney excretions, which begin around ED5^64^. Like amniotic fluid (6 ml), allantoic fluid reaches its maximum water content (≈ 8–14 ml) at ED12/13^63–65^. Concurrently, the CAM serves as the embryo’s primary respiratory surface during the latter half of incubation^64^. Avoiding disruption of the developing CAM through puncture is thus critical.

Despite initial findings suggesting a negative impact of the puncture procedure on hatch rates (Phase I study: *P* = 0.012; OR = 0.46; 95% CI: 0.23–0.87), subsequent results from Phase II study found no direct evidence that hatchability was impacted when conducted at the optimal ED7 timepoint. This variation may be attributed to differences in genetics as well as inter-operator variability and potential learning curves associated with the procedure being performed by different personnel in different laboratory settings. Such factors could influence the degree of mechanical trauma and contribute to the high resilience observed in Phase II.

We hypothesize that mid-embryonic death (ED7-ED12) represents the window most likely associated with direct puncture-induced trauma. While we observed a minor numerical increase in this phase during Phase I study (5% vs. 2%), the difference was not statistically significant (*P* = 0.113). In the Phase III study, isolated post-puncture deaths (ED8–ED11) were linked to visible hemorrhaging in the amnion. Nevertheless, no significant increase in cumulative embryonic mortality was observed (*P* = 0.369), suggesting that direct lethal injuries remain rare. Interestingly, the statistical significance in Phase I was primarily driven by the accumulation of both mid- and late-embryonic death. Late embryonic death typically occurs around ED19 due to factors such as malposition, membrane entrapment^67^ and failure to transition from allantois to pulmonary respiration^68^. Since a detailed investigation of these factors was beyond the scope of this study, the potential contribution of our puncture procedure to late embryonic mortality cannot be ruled out. Although the Phase II and Phase III studies indicated limited impact on hatchability when our method was performed at ED7, ongoing concerns from breeding companies regarding potential puncture-induced undesirable side effects (e.g. disturbed embryo development or contamination) have already led to the development of non-invasive *in ovo* sexing methods, as previously reviewed elsewhere^14^.

High-quality DNA obtained from *in ovo* fluids by WGA enabled the implementation of various assays, including fluorescence-based sexing and genotyping as well as multiplex PCR followed by gel electrophoresis. Our accuracy rates, ranging from 92 to 100% across all incubation days, are comparable to other established methods. This includes robotic, large-scale *in ovo* sexing system like that used used by PLANTegg GmbH^41^(≈ 99.5% accuracy at ED9) and published qPCR assays utilizing allantoic fluid DNA (95–100%)^42^. Crucially, the Pilot and Phase II studies clearly demonstrated that PCR success rates improved with increasing embryonic age, likely due to higher cell/DNA content in the samples. However, in the Phase I study, KASP assays required an 18% repeat rate due to initial clustering issues. We hypothesize that the allantoic fluid matrix, particularly high uric acid levels, may interfere with sensitive fluorescence signaling. Additionally, while WGA ensures sufficient DNA yields, it can introduce stochastic bias during early amplification cycles, impacting allelic discrimination, especially when using low-quantity or poor-quality template DNA from allantoic fluid. Further optimization, such as increased sample dilution or pre-amplification clean-up, might help mitigate these matrix effects and enhance diagnostic precision. While our method achieved high accuracy, minor discrepancies between sample and control results were observed (e.g., allantoic fluid samples being genotyped as male when control tissues were female). These were primarily attributed to inherent limitations such as human handling error during fluid collection or assay variability potentially caused by PCR inhibitors derived from the collected fluid.

## Conclusion

Our findings confirm that performing the invasive puncture and fluid extraction at ED7 has limited impact on embryo survival and hatchability, consistent with prior research^37,38,42^. Our four independent studies, which utilized various breeds and equipment, across multiple laboratories, demonstrate the method’s feasibility under diverse conditions. These results confirm that *in ovo* sexing and genotyping can be performed by smaller laboratories equipped with a PCR cycler or fluorometer, suggesting that this method can be rapidly adopted after brief training.

Implementation of this approach significantly helps to reduce the number of surplus animals, saving labor and resources while upholding the 3R principles. Furthermore, the potential application of similar fluid-sampling techniques in mammalian models, such as puncturing blastocysts in cattle and pigs^69^, highlights the broader relevance of this method. Ultimately, by integrating early *in ovo* identification with evolving ethical frameworks and improved pain management protocols^70–76^, researchers can leverage the chicken as a valuable animal model while ensuring the highest standards of animal welfare.

### Limitations of the study

Not all groups included in this study were brought to hatch, precluding an assessment of impact on hatchability. Additionally, follow-up monitoring of adult chickens (e.g. growth curves or laying performance) was not conducted. As sex determination in the Phase II study relied solely on vent sexing, it is not possible to ascertain whether discrepancies between KASP and vent sexing results are due to the sexer’s assessment, or to issues related to PCR or sample handling. Assessments for proper embryo development, including the ratio of embryo or yolk weight to total egg weight, were not part of this study. To enhance the statistical power of the data, future studies would benefit from testing a larger number of flocks and eggs across repeated trials including technical PCR replicates. In the absence of an absolute, error-free gold standard, it is difficult to definitively categorize the observed discordant results. Although control tissues were used, they are subject to similar risks of human handling errors, allelic dropout, or stochastic effects during amplification as the allantoic fluid samples themselves. Consequently, the reported accuracy rates should be interpreted as concordance levels between sample types rather than absolute diagnostic sensitivity.

## Supplementary Information

Below is the link to the electronic supplementary material.

Supplementary Material 1

## Data Availability

The datasets generated during and analysed during the current study are available from the corresponding author on reasonable request.
